# Supplementary material for: Human Wharton’s Jelly Mesenchymal Stem Cells Secretome Inhibits Human SARS-CoV-2 and Avian Infectious Bronchitis Coronaviruses
Source: Cells. 2022 Apr 21;11(9):1408. doi: 10.3390/cells11091408 (PMC9101656; doi:10.3390/cells11091408)
Supplement: Supplementary file 1 [file cells-11-01408-s001.zip › Supplementary figures.pdf]

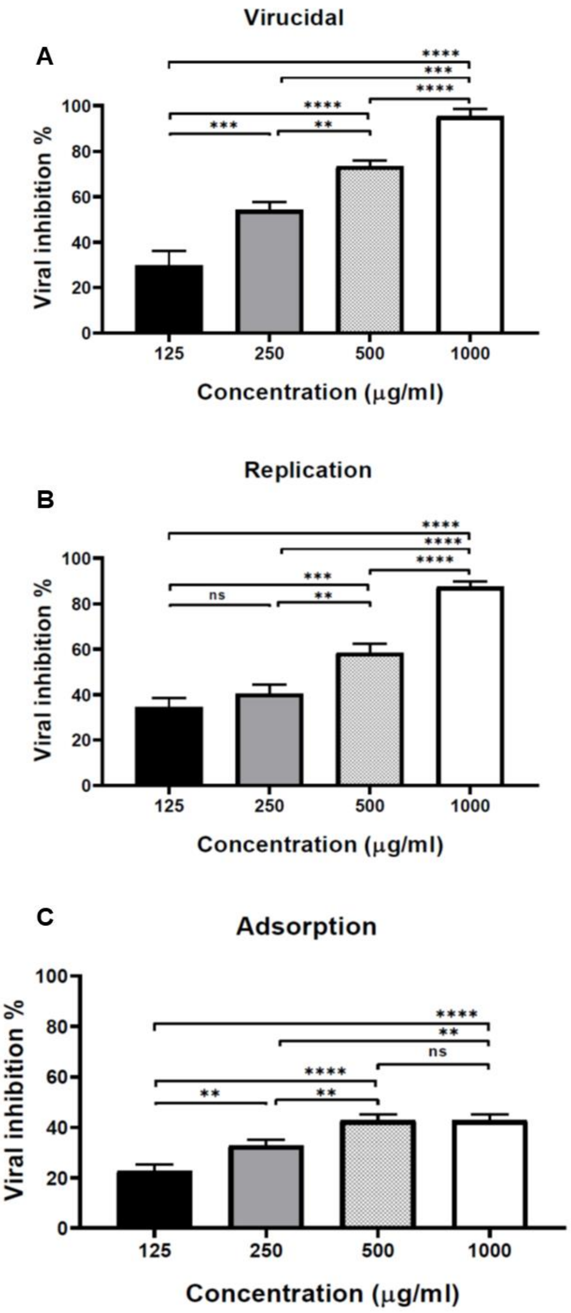

Figure S1. Comparison between different concentrations of WJ-MSC-S against SARS-CoV-2 using different antiviral assays. Virucidal **(A)**, anti-replication **(B)**, and anti-adsorption **(C)**. Results are shown as means ± SD of three independent experiments, each run in triplicate. *$p < 0.05$, **$p < 0.01$, ***$p < 0.001$, ****$p < 0.0001$ indicated the significant correlation among different concentrations of WJ-MSC-S.

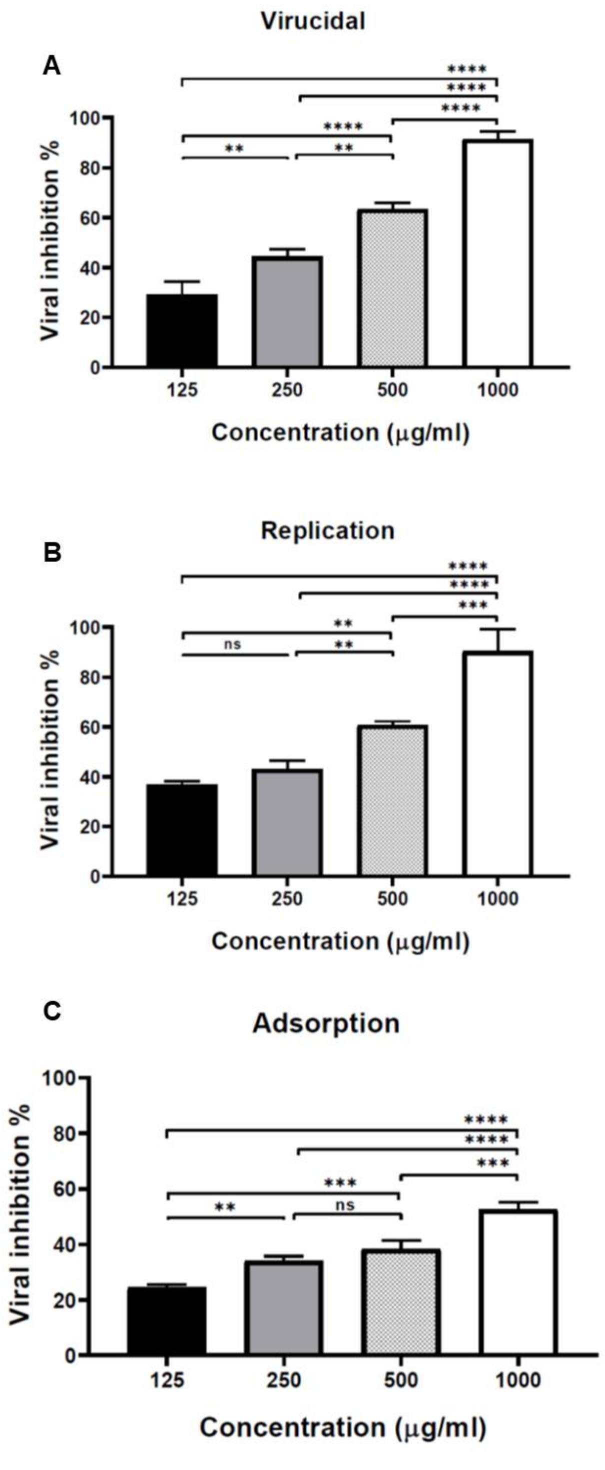

Figure S2. Comparison between different concentrations of WJ-MSC-S against IBV using different antiviral assays. Virucidal **(A)**, anti-replication **(B)**, and anti-adsorption **(C)**. Results are shown as means ± SD of three independent experiments, each run in triplicate. $^{*}p < 0.05$, $^{**}p < 0.01$, $^{***}p < 0.001$, $^{****}p < 0.0001$ indicated the significant correlation among different concentrations of WJ-MSC-S.
